# Repurposing of World-Approved Drugs for Potential Inhibition against Human Carbonic Anhydrase I: A Computational Study

**DOI:** 10.3390/ijms241612619

**Published:** 2023-08-09

**Authors:** Nannan Zheng, Wanyun Jiang, Puyu Zhang, Le Ma, Junzhao Chen, Haiyang Zhang

**Affiliations:** Department of Biological Science and Engineering, School of Chemistry and Biological Engineering, University of Science and Technology Beijing, Beijing 100083, China

**Keywords:** receptor-ligand interaction, metallo-enzymes, drug repurposing, molecular simulation

## Abstract

Human carbonic anhydrases (hCAs) have enzymatic activities for reversible hydration of CO_2_ and are acknowledged as promising targets for the treatment of various diseases. Using molecular docking and molecular dynamics simulation approaches, we hit three compounds of methyl 4-chloranyl-2-(phenylsulfonyl)-5-sulfamoyl-benzoate (84Z for short), cyclothiazide, and 2,3,5,6-tetrafluoro-4-piperidin-1-ylbenzenesulfonamide (3UG for short) from the existing hCA I inhibitors and word-approved drugs. As a Zn^2+^-dependent metallo-enzyme, the influence of Zn^2+^ ion models on the stability of metal-binding sites during MD simulations was addressed as well. MM-PBSA analysis predicted a strong binding affinity of −18, −16, and −14 kcal/mol, respectively, for these compounds, and identified key protein residues for binding. The sulfonamide moiety bound to the Zn^2+^ ion appeared as an essential component of hCA I inhibitors. Vina software predicted a relatively large (unreasonable) Zn^2+^–sulfonamide distance, although the relative binding strength was reproduced with good accuracy. The selected compounds displayed potent inhibition against other hCA isoforms of II, XIII, and XIV. This work is valuable for molecular modeling of hCAs and further design of potent inhibitors.

## 1. Introduction

Human carbonic anhydrases (hCAs) are a class of metallo-enzymes widely present in various tissues and cells, and they have a typical antiparallel β-sheet with a central fold that surrounds a Zn^2+^ ion that is essential for catalysis [1]. Their active sites are located in a large conical cavity with a Zn^2+^ ion at the bottom coordinated by three histidine residues and a water molecule/hydroxide ion [1,2] that catalyzes the reversible hydration of carbon dioxide [3,4,5,6,7]. The hCAs play an important role in a variety of physiological processes, such as acid–base balance [8], glaucoma [9], bone calcium metabolism [10], and nervous system development [11]. Therefore, they are acknowledged as potential drug targets for the treatment of diseases, such as epilepsy [12], hypertension [13], neuropathic pain [14], rheumatoid arthritis [15], and cancer [16].

The classification of hCAs depends on the similarity of genomic DNA and amino acid sequences, and 15 different types (isoforms) of hCAs have been identified [17,18], of which 12 (hCAs I–IV, hCAs VA–VB, hCAs VI–VII, hCA IX, and hCAs XII–XIV) are catalytically active and show similar active-site structures [19]. These isoforms vary in localization and tissue distribution and include cytoplasmic (I, II, III, VII, and XIII), membrane-bound (IV, IX, XII, and XIV), mitochondrial (VA and VB), and secretory (VI) isoforms [20,21,22]. The most common ones are hCA I, II, V, and IX, which serve as therapeutic targets for the treatment of many diseases [6]. For instance, hCA II is a potent target for glaucoma treatment and plays a role in intraocular production of bicarbonate [23], and its inhibitor sulfonamides have been clinically used as antiglaucoma drugs for decades [24]. The hCA VA and VB inhibitors can be used to fight obesity [25,26]. hCA IX and XII are specific for hypoxic tumor cells and are potential targets for cancer therapy [27,28]. The hCA I isoform was reported as a promising target for the treatment of retinal/brain edema [29]. The presence of extracellular hCA I, either within the blood–retinal barrier or the blood–brain barrier, can cause vasogenic edema [30]. Inhibiting extracellular hCA I can provide new therapeutic opportunities for the treatment of hemorrhagic retinal and brain edema [31]. Thus, it is of great clinical significance to study the biological function of hCA I and to design and discover effective hCA I inhibitors.

Recently, more and more research has focused on the discovery of hCA I inhibitors with high efficiency and selectivity [4,29,32,33,34,35,36]. Some natural products and chemically synthesized compounds were found to be capable of inhibiting hCA I and could be further optimized subsequently [37,38]. Several classes of hCA inhibitors (hCAIs) were reported: (i) sulfonylurea inhibitors, such as acetazolamide (AZM) and benzenesulfonic acid (BSA), which inhibited enzymatic catalysis via occupying the position of CO_2_ binding [39,40,41]; (ii) carboxylic acid inhibitors, which reduced the catalytic activity via binding water molecules/hydroxide ions [42,43,44]; and (iii) natural product inhibitors, which inhibited the catalytic activity by binding to hCA I and closing the substrate entrance to the active site [45,46,47]. These inhibitors have great potential for drug discovery and clinical treatment. Therefore, studies on the interaction of hCA I with potential inhibitors are of vital importance for understanding the biological function of hCA I and for developing related drugs.

Virtual screening is a drug discovery method using computational techniques and can be used to quickly and efficiently predict the binding affinity and inhibitory activity of compounds with targets [48]. Drug reuse is a promising strategy for exploring new uses of old drugs, accelerating the development of new drugs [49]. In this work, we present a systematic evaluation of existing hCA I inhibitors and a virtual screening of approved drug molecules via molecular docking, aiming to hit potent inhibitors against hCA I. After toxicity assessment, the complexes between hCA I and the selected inhibitors were subjected to molecular dynamics (MD) simulations. The molecular mechanics Poisson-Boltzmann surface area (MM-PBSA) analysis was then conducted to investigate the receptor-ligand interaction and identify key residues for ligand binding. The selectivity of hit compounds against hCA isoforms was addressed as well. This work has valuable implications for the design of potent inhibitors against hCAs.

## 2. Results and Discussion

### 2.1. Evaluation of Crystal Receptor-Ligand Complexes

**Figure 1:**
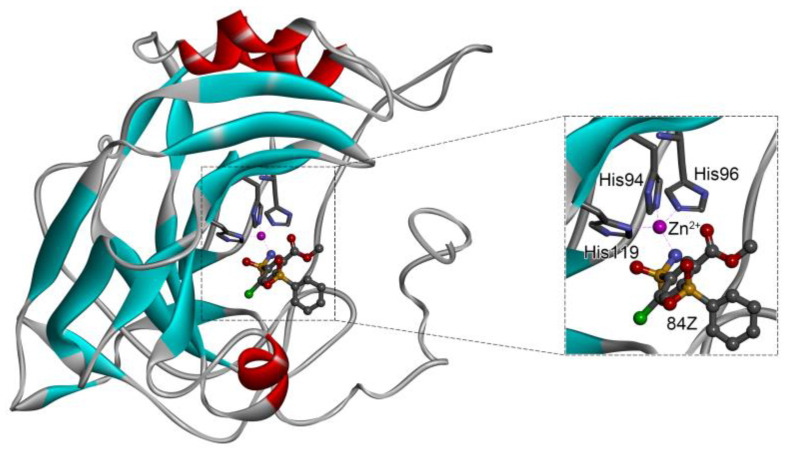
Crystal structure of hCA I in complex with the ligand 84Z (PDB ID: 7Q0D) and an enlarged view of the metal-binding center. Zn^2+^ coordinated with three histidine residues (His94, His96, and His119) and the N atom of 84Z. The protein is shown with a solid ribbon model colored by secondary structure types. The histidine residues are displayed with a stick model and the ligand with a scaled ball-and-stick model. The Zn^2+^ is represented by a pink ball.

In the crystal structure of hCA I (PDB code: 7Q0D), Zn^2+^ is bound to three protein residues (His94, His96, and His119), and the N atom of ligand (residue name: 84Z) coordinates with Zn^2+^ as well (Figure 1). For the 36 crystal structures of receptor-ligand complexes, Zn^2+^–ligand binding distances amounted to 1.73–2.21 Å, except for 3W6I and 2FW4, which had large distances of >5 Å (Table 1). Such short distances indicated a strong coordination between Zn^2+^ and ligands; in most cases, the metal coordination occurred with the sulfonamide moiety of the ligands. The binding affinities for these crystal complexes ranged from −8.0 (PDB code: 5GMM) to 1.9 (3W6I) kcal/mol.

Docking calculations predicted a Zn^2+^–ligand binding distance of 2.29–16.82 Å. About half of the ligands showed a distance of >3.5 Å, and the rest appeared to have more of a chance to coordinate with the Zn^2+^ ion. The predicted ligand poses displayed a root-mean-square deviation (RMSD) of 2.64 ± 1.57 Å from the crystal ones. The probability of finding the best pose was 85% on average. The docking showed that polmacoxib had the strongest binding affinity with hCA I (∆*E* = −9.0 kcal/mol), whereas the binding pose was not well reproduced compared with the crystal structure (Figure S1 in the Supplementary Materials), as indicated by a large binding distance (*d* = 3.41 Å) and root-mean-square deviation (RMSD = 3.95 Å), as shown in Table 1. The sulfonamide moiety of the ligand was positioned far away from the metal ion, and it did not coordinate with Zn^2+^ anymore (Figure S1).

**Figure 2:**
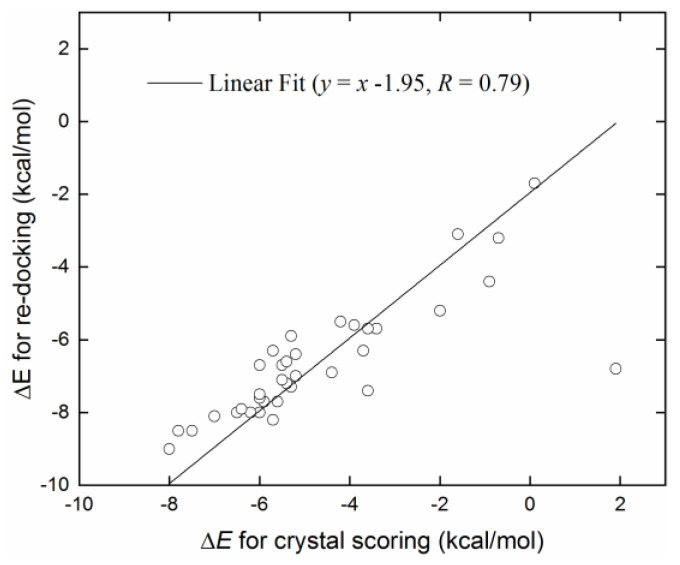
Comparison of binding affinities between crystal scoring and re-docking predictions for the 36 crystal receptor–ligand complexes in Table 1. The solid line is a linear fit of data points, and *R* is the Pearson correlation coefficient.

The Pearson correlation coefficient between the binding affinities from crystal scoring and re-docking was 0.79 (Figure 2), and the corresponding Spearman rank correlation coefficient was 0.85. These findings indicated that the Vina software showed a reasonable prediction for the relative binding strengths, although the Zn^2+^-involved binding details displayed discrepancies to some extent.

### 2.2. Virtual Screening of World-Approved Drugs against hCA I

Docking calculation of the 36 crystal receptor–ligand complexes showed that polmacoxib (residue name: 949; PDB code: 5GMM) has the strongest binding strength (∆*E* = −9 kcal/mol). Considering the metal-ligand coordination, we computed the distance between Zn^2+^ and ligand atoms (excluding C and H) for identification of possible coordination. After a virtual screening of world-approved drugs, 79 compounds with ∆*E* ≤ −9 kcal/mol and Zn^2+^–ligand binding distances ≤3.5 Å were selected for further evaluation (Table S1 in Supplementary Materials).

### 2.3. Toxicity Evaluation

Toxicity predictions were conducted using the online server ProTox-II for the selected 79 compounds from the world-approved drug set (Section 2.2) and the 36 ligands in the PDB database (Table 1), as listed in Tables S1 and S2 in the Supplementary Materials. Two different levels of toxicity (organ toxicity and toxicological endpoints) were considered. The compounds that were predicted to be toxic (marked Y) with a confidence of >50% for at least one type of toxicity were discarded. After the toxicity evaluation, we chose 11 compounds from 79 candidate inhibitors; 3 compounds had different charge states (neutral or +1 *e*). From the 36 crystal complexes, we also selected four ligands with relatively strong binding strengths and almost no toxicity, namely, polmacoxib, diart, 3UG, and 84Z. In total, we had 18 candidate inhibitors (Table 2) for subsequent MD simulation and MM-PBSA analysis.

### 2.4. Benchmark of Zn^2+^ Ion Models

**Figure 3:**
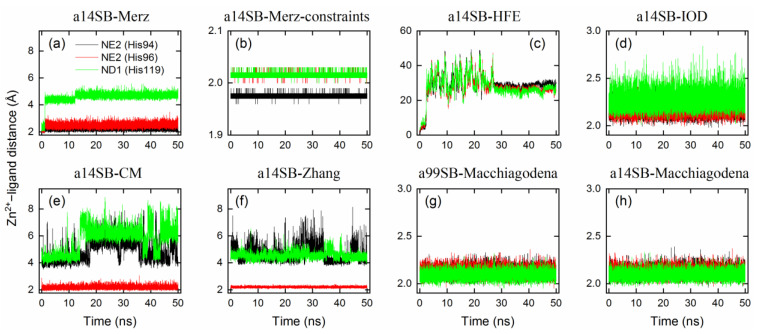
Zn^2+^–ligand binding distance as a function of simulation time for ligand-free hCA I using different protein force fields and ion models (**a**–**h**). Refer to Table 3 for the details of force-field parameters. Distances with the NE2 atom (black) of His94, the NE2 atom (red) of His96, and the ND1 atom (green) of His119 were monitored.

In order to maintain the stability of metal-binding networks (Figure 1) during MD simulations, a variety of 12-6 LJ Zn^2+^ ion models were benchmarked using the Amber ff14SB force field for hCA I. During 50 ns simulations, the Amber standard Zn^2+^ model by Merz, indicated by a legend of a14SB-MerZ in Figure 3a and Table 3, produced a stable coordination state between Zn^2+^ and His94/His96, whereas the Zn^2+^–His119 binding distance amounted to 4.7 ± 0.2 Å, implying the absence of metal coordination with His119 (Figure 3a). A threshold of 2.5 Å is often used to check whether a metal coordination exists or not. Using a harmonic potential to constrain the Zn^2+^–N distance offers a solution, as indicated by tiny fluctuations of the Zn^2+^–ligand distance (Figure 3b). The HFE set by Li et al. produced totally disrupted metal networks with a binding distance of > 25 Å (Figure 3c and Table 3), and the coordination numbers of Zn^2+^ were largely affected as well (Table 3). The IOD set by Li et al. targeted the ion–water oxygen distance and hence gave a reasonable metal-binding network, while the Zn^2+^–His119 distance appeared slightly larger than that in the crystal state (Figure 3d and Table 3). The CM set by Li et al. (Figure 3e) and our model (Figure 3f) showed a similar performance and only generated one coordinated state with His96.

**Figure 4:**
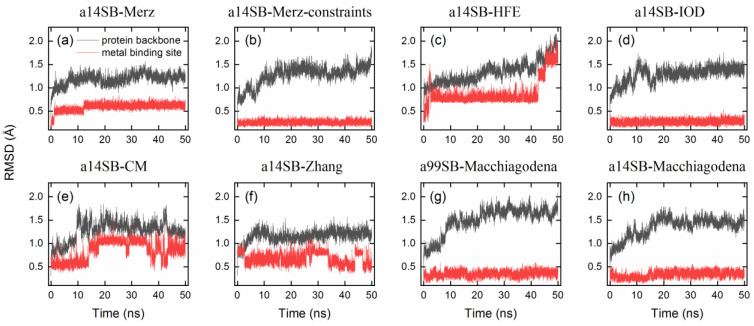
RMSDs of the hCA I protein backbones (black) and metal-binding sites (red) from crystal structures as a function of simulation time for ligand-free hCA I using different protein force fields and ion models (**a**–**h**). Refer to Table 3 for the details of force-field parameters.

With a modification of His residues using the Amber 99SB-ILDN force field, the Zn^2+^ model by Macchiagodena et al. reproduced the crystal metal-binding site with good accuracy (Figure 3g and Table 3). A very similar performance was observed when using the Amber ff14SB force field (Figure 3h), while it yielded a slightly smaller RMSD (1.5 Å) for the protein backbone than that obtained with the Amber 99SB-ILDN force field (1.7 Å, Table 3). RMSDs of the protein backbone and metal-binding site (i.e., Zn^2+^ and the three bound His residues) are given in Figure 4 and Table 3. If the metal-binding site was not maintained well, the backbone of protein hCA I would display a large RMSD from the crystal structure (Figure 4). Based on these findings, we chose the Zn^2+^ model by Macchiagodena et al. and the Amber ff14SB force field (note that force-field modifications of His residues were also needed) for the following MD simulations of receptor–ligand complexes.

The modified protein parameters for the Amber 14SB force field were also tested for use with the Amber standard Zn^2+^ model by Merz [50] and the HFE, IOD, and CM models by Li et al. [51], as well as with the model by Zhang et al. [52]. Surprisingly, all of these five models produced a Zn^2+^–ligand binding distance of <2.5 Å (Figure S2 in the Supplementary Materials). However, the Merz, HFE, and CM models yielded much smaller Zn^2+^–ligand binding distances of 1.7–1.8 Å compared with the crystal structure (Figure S2 and Table 3). The IOD and Zhang models gave relatively reasonable binding distances of ca. 2.08 and 2.16 Å, respectively. During the 50 ns simulation, the Merz, HFE, and Zhang models failed to maintain the structural stability of the protein backbone and/or metal-binding sites, while the IOD and CM models showed a good performance, as indicated by the RMSE values in Figure S3 in the Supplementary Materials. The IOD model appeared to have a good transferability for the tested protein system. For a general purpose, based on our test, the protein force-field modifications proposed by Macchiagodena et al. are strongly recommended to be used with the Zn^2+^ model designed by the same author [53,54], as in our following MD simulations.

### 2.5. MD Simulation of hCA I–Inhibitor Complexes and Binding Energy Calculations

Initial configurations of hCA I–inhibitor complexes were taken from crystal structure or docking predictions. Based on the selection criteria, the 18 compounds in Table 2 should be coordinated with Zn^2+^ ions or have more of a chance for coordination.

After the 50 ns simulation, there were 11 compounds that did not coordinate with Zn^2+^ ions and/or went further away from the substrate-binding pocket of hCA I, as indicated by a comparison of the Zn^2+^–ligand binding distance (*d*_bound_) and the distance between the centroid of the metal-binding site and ligand (*d*_ML_), as shown in Table 4. For instance, diart coordinated with Zn^2+^ with a distance of 1.98 Å in the crystal state (PDB code: 7ZL5), while the complex structure was not stable and the ligand escaped from the binding pocket (Table 4). The remaining seven compounds were then used for MM-PBSA analysis.

For the crystal structure, 84Z, polmacoxib, and 3UG preferred to offer the N atom in the sulfonamide moiety for coordination with the Zn^2+^ ion. During MD simulation, however, the O atom in the sulfonamide moiety preferred the coordination over the N atom for polmacoxib and 84Z (Table 4). A similar finding was observed for the inhibitor of cyclothiazide (selected from world-approved drugs). Both N and O atoms might coordinate with Zn^2+^ ions simultaneously, as observed for the inhibitor bhft.

Table 5 lists the MM-PBSA analyses of energy decomposition for the seven compounds whose one or two atoms coordinated with the Zn^2+^ ion. 84Z, cyclothiazide, and 3UG showed a strong binding with hCA I with binding energies of −18, −16, and −14 kcal/mol, respectively, and these three compounds were finally selected as potential inhibitors against hCA I. Compared with these compounds, bhft and ketoprofen glucuronide yielded much more favorable MM contributions (van der Waals ∆*E*_vdW_ + electrostatic ∆*E*_elec_); however, due to the large unfavorable solvation contributions, a relatively weak binding was observed (Table 5). Polmacoxib and bemetizide displayed a very weak binding with hCA I, as indicated by a near-zero binding energy (Δ*E*_bind_), as shown in Table 5.

**Figure 5:**
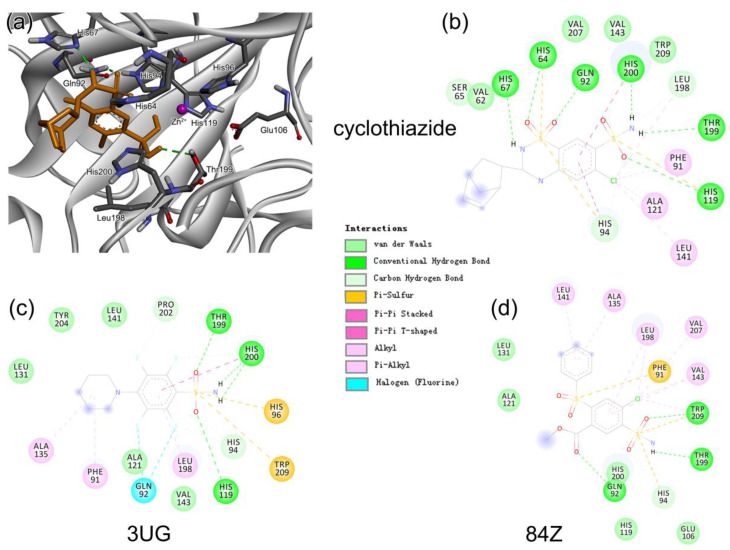
(**a**) Representative binding mode of hCA I with cyclothiazide from MD simulations and (**b**) the corresponding 2D diagram of receptor–ligand interactions. (**c**,**d**) show 2D ligand interactions with the receptor hCA I for 3UG and 84Z, respectively.

Representative 3D and 2D diagrams of receptor–ligand interactions for the hit compounds of 84Z, cyclothiazide, and 3UG are presented in Figure 5. A variety of interactions were detected for ligand interactions, such as hydrogen bonds, π-sulfur interactions between aromatic residues and the S atom of the sulfonamide group, and π-π interactions between aromatic rings. The 3UG compounds contained F atoms, offering halogen interactions with the protein residue Gln92 (Figure 5). For cyclothiazide, six hydrogen bonds were detected with hCA I residues of His64, His67, Gln92, His119, Thr199, and His 200 (Figure 5a,b).

### 2.6. Identification of Key Residues for Receptor–Inhibitor Interactions

**Figure 6:**
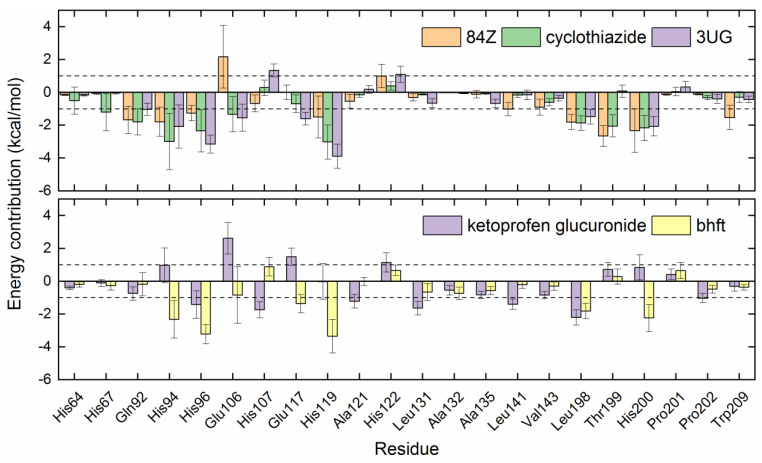
Energy contribution per residue to the binding of hCA I with the potential inhibitors of 84Z, cyclothiazide, 3UG, ketoprofen glucuronide, and bhft. The residues with a contribution of ≥1 kcal/mol for at least one inhibitor are presented, and dashed lines indicate a contribution of ±1 kcal/mol.

To identify key residues of hCA I for ligand binding, binding energies obtained from MM-PBSA analysis were further decomposed into per-residue contributions. For the compounds of 84Z, cyclothiazide, 3UG, ketoprofen glucuronide, and bhft, we identified 22 amino acid residues with a contribution of ≥1 kcal/mol to the binding of at least one inhibitor, as shown in Figure 6. Zn^2+^-binding His residues (His94, His96, and His119) displayed a large favorable contribution of <−2 kcal/mol, except for ketoprofen glucuronide with a contribution of ~1 kcal/mol. Glu106, located in the deep bottom of the substrate-binding pocket (Figure 5a), disfavored the binding with 84Z and ketoprofen glucuronide, while it had favorable contributions with the other three inhibitors. Hydrophobic residues, such as Ala, Leu, Val, and Thr residues, tended to favor the binding via van der Waals interactions (Figure 5 and Figure 6). 

Detailed energies per residue for van der Waals and electrostatic contributions are given in Tables S3–S7 in the Supplementary Materials for the five compounds. Zn^2+^ was regarded as a receptor residue. Zn^2+^ offered favorable Δ*E*_MM_ contributions; however, due to the large solvation part, it appeared to disfavor the ligand binding.

### 2.7. Selectivity against hCA Isoforms

Binding affinities between the hit compounds of 84Z, cyclothiazide, and 3UG and the 12 isoforms (with enzymatic activity) of hCAs were predicted from 50 docking runs and are listed in Table 6. The results revealed that the hit compounds appeared to be selective inhibitors against hCA I, as indicated by a strong binding affinity, while they might be potent inhibitions against hCA II, XIII, and XIV. Further high-accuracy computational and/or in vitro experimental tests were necessary to test the inhibition and selectivity activity of the hit compounds. One can also crystallize and solve the structure of hCA I complexes with these potential inhibitors, providing a proof that these compounds really bind to the receptor at a molecular level.

## 3. Materials and Methods

### 3.1. Docking Protocol

#### 3.1.1. Receptor Preparation

The 3D structure of receptor hCA I was taken from the Protein Data Bank database (PDB code: 7Q0D) with a high resolution of 1.24 Å [19], in which it formed complexes with a ligand (residue name: 84Z). There existed more than 40 ligands bound to hCA I (https://www.rcsb.org/groups/sequence/polymer_entity/P00915; accessed on 30 June 2023), and we ignored the ligands that were singly charged ions (like I^−^) or which contained unusual elements, such as Au, Cu, and Se. The resulting 36 complexes were evaluated as well.

#### 3.1.2. Ligand Preparation

The world-approved drugs (5903 compounds) were downloaded from the ZINC 15 [55] database (https://zinc.docking.org/substances/subsets/world; accessed on 30 June 2023). Some compounds had multiple charge states or isoforms at different pH levels, resulting in 7658 drugs. Due to docking parameters for B, Si, and Sn atoms being missing, we ignored 22 drugs and had 7636 compounds in total for use as ligands. The 36 ligands from the PDB database (as mentioned in Section 3.1.1) did not belong to the world-approved drug database, and they were reported with potential inhibition against hCA I.

#### 3.1.3. Docking Calculation

AutoDock Vina software (version 1.1.2) [56] was used for the docking calculations. The protein 7Q0D was used as the receptor for virtual screening of inhibitors. The center of searching space (30 × 30 × 30 Å^3^) in the docking was set to the geometric center of its ligand (i.e., 84Z). Similarly, the 36 crystal hCA I–ligand complexes were also scored and re-docked for evaluation of ligand binding affinities and verification of the docking protocol.

### 3.2. Toxicity Prediction

Toxicities of selected compounds were predicted by a web server of ProTox-II (https://tox-new.charite.de/protox_II; accessed 30 June 2023) [57]. We considered two levels of toxicity: organ toxicity (hepatotoxicity) and toxicological endpoints (mutagenicity, carcinotoxicity, cytotoxicity, and immunotoxicity).

### 3.3. Simulation Protocol

Molecular dynamics (MD) simulations were carried out to capture the structural stability of receptor-ligand interactions. As the performance of ion models (like Zn^2+^) in the simulation of metallo-enzymes likely differed from case to case [58], we first benchmarked different Zn^2+^ models to check whether the metal-binding site can be well maintained during the simulation of ligand-free hCA I systems. Then, we chose a reasonable ion model to simulate the receptor-ligand complexes.

#### 3.3.1. Ligand-Free Systems

A variety of 12-6 Lennard–Jones (LJ) Zn^2+^ models were tested: the Amber standard ion model by Merz [50] and three sets of HFE, IOD, and CM models by Li et al. [51], as well as the models developed by us [52] and by Macchiagodena et al. [53,54]. Li’s HFE set targeted the hydration free energy of ions, the IOD set targeted ion–water oxygen distance, and the CM set was a compromised model for both properties of HFE and IOD. Our model was designed to target both properties. The Zn^2+^ model by Macchiagodena et al. was developed for use with the Amber ff99SB-ILDN force field [59]; when using this model, additional modifications of force-field parameters of protein residues (such as Zn^2+^-binding His, Cys, Asp, and Glu) were needed. Following the work by Macchiagodena et al., we made slight modifications to the Amber ff14SB force field [60] for consistency with this ion model, and the corresponding topological and parameter files are given in the Supplementary Materials. Neutral histidine (His) residues have two tautomeric states of Hid and Hie with a proton connected to δ- and ε-nitrogens, respectively. For the three key His residues in the metal-binding site (Figure 1), we used the Hid state for His94 and His96 and the Hie state for His119. Protonation states of other titratable residues were selected automatically at neutral pH by the GROMACS utility of “gmx pdb2gmx” [61].

Crystal water molecules close to protein atoms (within 5 Å) were retained, and the apo form of hCA I was placed in a simulation box with a length of ~60 Å. The box was then filled with water molecules, and Na^+^ and Cl^−^ ions were inserted into the box for neutralizing the system and obtaining a salt concentration of 0.15 mol/L. The system contained 1 protein, 20 Na^+^, 22 Cl^−^ ions, and ca. 5800 water molecules. After energy minimization, we conducted 100 ps NVT and 400 ps NPT equilibrations, followed by 50 ns production simulations at NPT (P = 1bar and T = 298.15 K). The detailed MD protocol was presented in our previous work [62,63]. All of the MD simulations were carried out using the GROMACS software (version 2018.4) [61].

#### 3.3.2. Receptor–Ligand Complexes

The General Amber Force Field (GAFF) [64] was used for the selected ligands. We optimized the ligand structure at HF/6-31G* in gas phase via Gaussian 09 software [65] and then computed the restrained electrostatic potential (RESP) charges of the ligands. Receptor–ligand complexes were taken from available crystal structures or, if not, docking predictions. The simulation setup for receptor–ligand complexes was similar to what was mentioned in Section 3.3.1.

### 3.4. MM-PBSA Analysis

The last 20 ns of simulation trajectories for receptor–ligand complexes were used for the molecular mechanics Poisson–Boltzmann surface area (MM-PBSA) analysis to compute the binding energy (∆*E*_bind_) and identify key residues for ligand interactions. The Zn^2+^ ion was regarded as a receptor residue. We first stripped water molecules and Na^+^ and Cl^−^ ions from the trajectory and saved it to a new trajectory with an interval of 100 ps. We therefore had 201 snapshots of receptor–ligand complexes for the MM-PBSA analysis. The binding energy (∆*E*_bind_) from such analysis is composed of van der Waals (∆*E*_vdW_) and electrostatic (∆*E*_elec_) contributions as well as polar (∆*G*_pola_) and nonpolar (∆*G*_nonpolar_) solvation contributions; the first two are known as the molecular mechanics (${∆E}_{MM}$) part, while the last two are the solvation part (${∆G}_{sol}$), as listed in Equation (1).

(1) $${∆E}_{bind}={∆E}_{MM}+{∆G}_{sol}={∆E}_{vdw}+{∆G}_{elec}+{∆G}_{polar}+{∆G}_{nonpolar}$$

With entropy estimation, one can obtain the known binding free energies. Such estimation is computationally expensive in general, and its value is highly dependent on the configuration sampling and on the choice of the methods used. Here, we did not consider the entropy contribution to the binding. The analysis and energy decomposition per residue were performed by the “gmx_MMPBSA” package [66,67]. For the per-residue energy decomposition, we chose to output all residue contributions, and the other parameters for the MM-PBSA analysis were set by default.

### 3.5. Inhibition against hCA Isoforms

The human carbonic anhydrase family is known to have 15 isoforms [17,18] with similar 3D structures, of which three members (hCA VIII, X, and XI) have almost no enzymatic activity [68,69]. In order to evaluate the selectivity of the chosen inhibitors based on hCA I, we ran docking calculations using the other 11 isoforms as a receptor for comparison. The receptor conformations were taken from experimentally determined 3D structures in the PDB archive or, if not, the computed models in the AlphaFold Protein Structure database (https://alphafold.ebi.ac.uk/; accessed on 30 June 2023) [70].

## 4. Conclusions

Using molecular docking, toxicity prediction, and molecular dynamics simulation techniques, we identified three compounds from existing ligands in the PDB database and world-approved drugs, namely, methyl 4-chloranyl-2-(phenylsulfonyl)-5-sulfamoyl-benzoate (84Z for short), cyclothiazide, and 2,3,5,6-tetrafluoro-4-piperidin-1-ylbenzenesulfonamide (3UG for short). These compounds likely acted as potent inhibitors against human carbonic anhydrase I (hCA I) or other hCA isoforms of hCA II, XIII, and XIV. 

A sulfonamide moiety appeared to be an essential component of hCA inhibitors, and its N or O atoms preferred to coordinate with the Zn^2+^ ion in the bottom of the hCA I substrate-binding pocket. Unfortunately, Vina docking failed to predict such coordination, although it was indeed able to reproduce the relative binding strength. Protein and Zn^2+^ force-field models are of vital importance to maintain the metal-binding networks, and preliminary tests of different models are therefore needed for molecular modeling of metallo-enzymes such as hCAs. We believe this work has valuable implications for computational simulations of hCAs and rational design of their inhibitors.

## Figures and Tables

**Table 1:** Binding affinities (∆*E*, kcal/mol) and Zn^2+^–ligand coordination distances (*d*, Å) for crystal structures of hCA I in complex with different ligands and docking predictions.

Res Name	PDB ID	Ligand Name	Molecular Structure	Crystal Complex		Docking Predictions			
				∆*E*	*d*	∆*E*	*d*	RMSD	Prob
949	5GMM	Polmacoxib	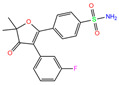	−8.0	1.95	−9.0	3.41	3.95	1
TOR	3LXE	Topiramate	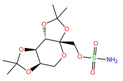	−7.5	2.00	−8.5	2.73	4.35	1
GZE	6I0J	GZE	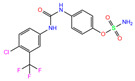	−7.8	2.00	−8.5	2.55	0.73	0.7
V14	5E2M	V14	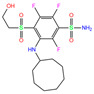	−5.7	1.95	−8.2	2.91	1.36	1
BZW	6EVR	BZW	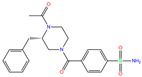	−7.0	1.94	−8.1	4.96	1.34	1
IWE	7ZL5	Diart	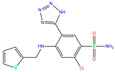	−6.0	1.98	−8.0	2.86	1.12	1
N19	6EX1	N19	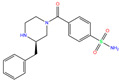	−6.2	1.92	−8.0	4.88	1.95	1
D3B	6FAF	D3B	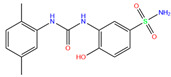	−6.5	1.96	−8.0	4.74	1.23	0.3
3UG	4WUQ	3UG	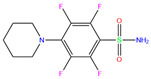	−6.4	1.84	−7.9	2.92	1.25	1
CJK	6F3B	CJK	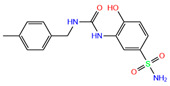	−5.6	1.93	−7.7	4.58	2.06	1
O5N	6XZY	O5N	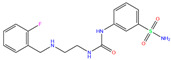	−5.9	1.84	−7.7	3.15	3.33	0.8
EON	6FAG	EON	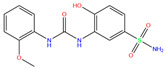	−6.0	1.90	−7.6	2.47	2.20	0.6
O5H	6XZX	O5H	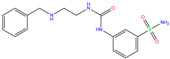	−6.0	1.90	−7.5	2.96	2.50	0.4
84Z	7Q0D	84Z	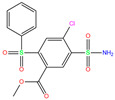	−3.6	1.91	−7.4	3.96	2.43	0.9
O4Z	6XZE	O4Z	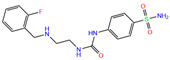	−5.3	1.95	−7.3	4.11	4.09	1
O5K	6XZS	O5K	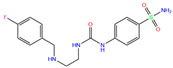	−5.4	1.89	−7.2	4.24	4.73	0.7
3TV	4WR7	3TV	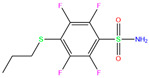	−5.5	1.84	−7.1	3.12	1.34	1
O55	6XZO	O55	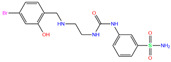	−5.2	1.93	−7.0	3.07	7.43	0.9
7TI	7PLF	Clorsulon	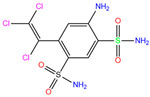	−4.4	1.94	−6.9	2.85	3.23	1
FLB	3W6I	FLB	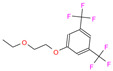	1.9	5.06	−6.8	7.31	3.19	1
O5T	6Y00	O5T	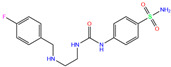	−6.0	1.92	−6.7	4.99	1.57	0.1
M25	2NMX	M25	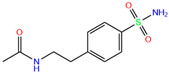	−5.5	2.19	−6.7	2.31	2.72	1
M29	2NN7	M29	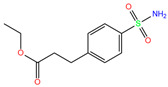	−5.4	2.11	−6.6	2.36	3.22	1
M28	2NN1	M28	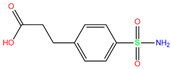	−5.2	2.21	−6.4	2.61	2.67	1
AZM	3W6H	Acetazolamide	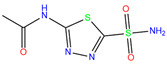	−3.7	2.17	−6.3	2.29	2.57	0.1
MZM	1BZM	Methazola-mide	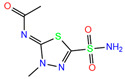	−5.7	1.99	−6.3	2.88	1.32	0.6
AZM	1AZM	Acetazola-mide	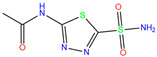	−5.3	2.01	−5.9	3.68	2.22	1
3UF	4WUP	3UF	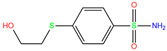	−3.4	1.74	−5.7	16.21	15.72	1
FO9	6G3V	Famotidine	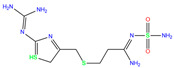	−3.6	1.95	−5.7	16.82	15.52	1
AAS	1CZM	3-Amabs	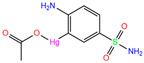	−3.9	1.86	−5.6	2.42	2.24	1
GZH	6I0L	GZH	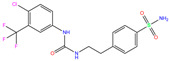	−4.2	1.92	−5.5	3.96	1.84	1
HIS	2FW4	d-histidine	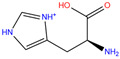	−2.0	29.79	−5.2	16.13	16.13	1
PPF	2IT4	Foscarnet	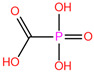	−0.9	1.73	−4.4	13.08	15.59	1
EDO	1JV0	Ethylene glycol	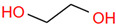	−0.7	1.82	−3.2	4.59	6.04	0.8
BCT	1HCB	Carbonate	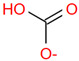	−1.6	1.78	−3.1	16.17	12.89	1
EDO	1J9W	Ethylene glycol	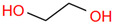	0.1	1.75	−1.7	14.26	13.57	1

Crystal structures of hCA I complexes with ligands were taken from the PDB database (ligand residue name and PDB ID) and were scored (∆*E*) by Autodock Vina software. The Zn^2+^–ligand coordination distance (*d*) is the distance between the Zn^2+^ ion and the corresponding coordinated atom of the ligand. Ten docking runs with random seeds (i.e., re-docking) were carried out to predict the ligand binding poses; averaged values of both properties (∆*E* and *d*) for the best poses in the docking predictions are given for comparison with the crystal structures. Root-mean-square deviations (RMSD) of the best binding poses from the crystal ones and the probabilities (Prob) for finding the best poses are listed in the last two columns. Polmacoxib, diart, 3UG, and 84Z were selected for further MD simulation and analysis.

**Table 2:** Selected compounds with potent inhibition against hCA I and relatively low toxicity.

ZINC ID	Name	Molecular Structure	*q*	∆*E*_dock_	Toxicity				
					Dili	Carcino	Immuno	Mutagen	Cyto
ZINC000011681563	Netupitant	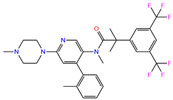	0	−9.9	N(66)	N(71)	N(78)	N(73)	N(64)
ZINC000011681563	Netupitant	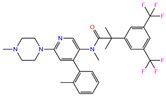	1	−9.9	N(66)	N(71)	N(78)	N(73)	N(64)
ZINC000000601301	Bhft	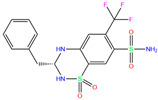	0	−9.8	N82)	N(71)	N(89)	N(84)	N73)
ZINC000205224698	Pronetupitant	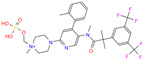	1	−9.8	N(66)	N(58)	N(53)	N(55)	N(61)
ZINC000027990463	Lomitapide	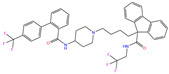	1	−9.7	N(78)	N(61)	N(70)	N(58)	N(77)
ZINC000021981256	8-hydroxymirtazapine	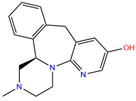	1	−9.6	N(83)	N(66)	N(96)	N(61)	N(59)
ZINC000003816514	Rolapitant	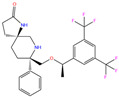	0	−9.6	N(83)	N(66)	N(93)	N(67)	N(75)
ZINC000003816514	Rolapitant	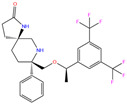	1	−9.6	N(83)	N(66)	N(93)	N(67)	N(75)
ZINC000022034381	Lidoflazine	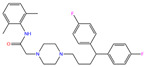	0	−9.5	N(79)	N(70)	N(97)	N(78)	N(67)
ZINC000031417974	Ketoprofen glucuronide	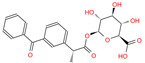	−1	−9.3	N(71)	N(68)	N(85)	N(85)	N(82)
ZINC000002570882	4-hydroxyalprazolam	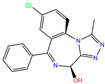	0	−9.2	N(52)	N(67)	N(97)	N(74)	N(70)
ZINC000000607726	Bemetizide	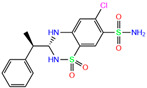	0	−9.2	N(89)	N(76)	N(88)	N(92)	N(81)
ZINC000022034381	Lidoflazine	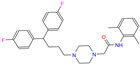	1	−9.2	N(79)	N(70)	N(97)	N(78)	N(67)
ZINC000100036907	Cyclothiazide	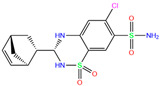	0	−9.1	N(91)	N(74)	N(91)	N(88)	N(73)
ZINC000000589683	Polmacoxib	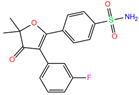	0	−9.0	N(53)	N(62)	N(89)	N(70)	N(67)
ZINC0000005843546	Diart	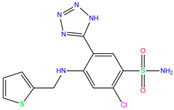	0	−8.0	N(59)	N(53)	N(95)	N(69)	N(62)
ZINC0000263621146	3UG	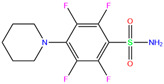	0	−7.9	N(66)	N(58)	N(99)	N(81)	N(69)
	84Z	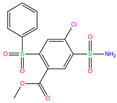	0	−7.4	N(65)	N(67)	N(99)	N(75)	N(70)

ZINC ID is the compound ID recorded in the ZINC database. The first 14 inhibitors were selected from the world-approved drug database with a strong binding affinity and a Zn^2+^–ligand binding distance within 3.5 Å, and the remaining 4 compounds were taken from the PDB database where they formed inclusion complexes with the receptor hCA I (as mentioned in Table 1). *q* is the net charge of inhibitors. ∆*E*_dock_ is the binding affinity (kcal/mol) by docking predictions. The toxicity was predicted using ProTox-II; dili is short for hepatotoxicity, carcino for carcinogenicity, immuno for immuno-toxicity, mutagen for mutagenicity, and cyto for cytotoxicity. N indicates inactive and Y active. The number given in parenthesis is the confidence (%) for the toxicity prediction.

**Table 3:** Time-averaged RMSDs for protein backbones and metal-binding sites, Zn^2+^–His binding distances, and the coordination numbers of Zn^2+^ ions from the last 20 ns MD simulations using different protein force fields and ion models.

Legend	Force Field	Zn^2+^ Model				RMSD_backbone_	RMSD_metal_	Zn^2+^–Ligand Distance (Å)			CN_Zn_
		Parameter Set	*R*	*ε*	Ref	(Å)	(Å)	NE2 (H94)	NE2 (H96)	ND1 (H119)	
a14SB-Merz	ff14SB	Merz	1.1	0.0125	[50]	1.25 ± 0.08	0.62 ± 0.05	2.25 ± 0.14	2.54 ± 0.19	4.71 ± 0.17	4.4
a14SB-Merz-constraints	ff14SB	Merz (constraints)	1.1	0.0125	[50]	1.37 ± 0.12	0.26 ± 0.04	1.98 ± 0.01	2.02 ± 0.01	2.02 ± 0.01	6.0
a14SB-HFE	ff14SB	HFE set by Li et al.	1.175	0.00071558	[51]	1.55 ± 0.19	1.05 ± 0.37	28.57 ± 1.45	26.31 ± 1.25	25.69 ± 1.54	3.0
a14SB-IOD	ff14SB	IOD set by Li et al.	1.395	0.014917	[51]	1.38 ± 0.08	0.28 ± 0.05	2.17 ± 0.07	2.18 ± 0.07	2.28 ± 0.09	6.0
a14SB-CM	ff14SB	CM set by Li et al.	1.271	0.00330286	[51]	1.32 ± 0.12	0.91 ± 0.16	5.00 ± 0.80	2.20 ± 0.13	6.10 ± 0.83	6.0
a14SB-Zhang	ff14SB	Zhang et al.	0.5152	295.5289	[52]	1.21 ± 0.08	0.65 ± 0.14	4.57 ± 0.59	2.20 ± 0.05	4.55 ± 0.28	7.0
a99SB-Macchiagodena	ff99SB-ILDN	Macchiagodena et al.	1.4561	0.0125	[53,54]	1.69 ± 0.10	0.36 ± 0.07	2.12 ± 0.05	2.12 ± 0.05	2.09 ± 0.04	6.0
a14SB-Macchiagodena	ff14SB	Macchiagodena et al.	1.4561	0.0125	[53,54]	1.46 ± 0.09	0.36 ± 0.06	2.12 ± 0.05	2.12 ± 0.05	2.09 ± 0.04	6.0
crystal-7q0d								1.97	2.02	2.02	4.0

Amber force fields of ff14SB and ff99SB-ILDN were used for MD simulations of the apo form of hCA I. When using the Zn^2+^ model by Macchiagodena et al., additional modifications were needed for the force-field parameters of Zn^2+^ binding amino acids (i.e., His residues in this work). The legends used in the subsequent figures are given in the first column. The ion modes tested were modeled by 12-6 LJ potential with two parameters of *R* and *ε*. In the crystal structure (crystal-7q0d), Zn^2+^ bound to three protein residues of His94, His96, and His119 and to the ligand (CN_Zn_ = 4); coordinated water molecules were not detected.

**Table 4:** Distance (Å) between the centroid of the metal-binding site and ligand (*d*_ML_) and between Zn^2+^ and the bound atom of the ligand (*d*_bound_) in the initial structure before the MD simulations and time-averaged distances from the last 30 ns simulations.

Ligand Name	*q*	*d*_ML_		Ligand Atom	*d*_bound_	
		Initial	MD		Initial	MD
Netupitant	0	1.04	1.59 ± 0.11	F6	2.95	12.97 ± 2.24
Netupitant	1	1.14	2.93 ± 0.56	F6	3.14	27.7 ± 5.63
Bhft	0	0.9	0.92 ± 0.03	O1	2.53	2.33 ± 0.15
				N1	4.73	2.31 ± 0.12
Pronetupitant	0	1.12	2.09 ± 0.34	F6	2.83	17.81 ± 5.85
Lomitapide	1	1.15	1.91 ± 0.38	F2	2.82	16.57 ± 4.75
8-hydroxymirtazapine	1	0.92	2.58 ± 0.71	O1	2.55	27.58 ± 6.54
Rolapitant	0	0.95	1.61 ± 0.11	F6	3.47	11.82 ± 1.98
Rolapitant	1	0.94	1.49 ± 0.09	F5	2.67	11.01 ± 1.41
Lidoflazine	0	0.96	1.42 ± 0.06	F1	2.88	4.37 ± 0.24
Ketoprofen glucuronide	−1	0.99	1.08 ± 0.02	O4	3.22	2.08 ± 0.06
4-hydroxyalprazolam	0	0.85	1.95 ± 0.04	N1	2.92	14.79 ± 0.63
Bemetizide	0	0.81	0.85 ± 0.03	N2	2.30	2.31 ± 0.12
Lidoflazine	1	0.96	1.34 ± 0.13	F1	2.80	4.63 ± 0.30
Cyclothiazide	0	0.85	0.81 ± 0.02	N1	2.47	4.43 ± 0.24
				O2	2.60	2.44 ± 0.21
Polmacoxib	0	0.89	0.91 ± 0.04	N1	1.95	4.62 ± 0.19
				O3	2.88	2.33 ± 0.17
Diart	0	0.81	1.32 ± 0.15	N5	1.98	10.39 ± 1.46
3UG	0	0.82	0.83 ± 0.02	N1	1.84	2.30 ± 0.10
84Z	0	0.84	0.86 ± 0.02	N1	1.91	4.39 ± 0.22
				O3	2.99	2.29 ± 0.13

**Table 5:** Decomposition of binding energy (kcal/mol) for selected inhibitors against hCA I from the MM-PBSA analysis of the last 30 ns simulation trajectories.

Compound	*q*	Δ*E*_vdW_	Δ*E*_elec_	Δ*E*_MM_	Δ*G*_polar_	Δ*G*_nonpolar_	Δ*E*_bind_
84Z	0	−34.25 ± 1.17	−43.15 ± 2.00	−77.4 ± 2.15	63.13 ± 1.51	−3.57 ± 0.03	−17.84 ± 1.80
Cyclothiazide	0	−39.51 ± 1.27	−38.52 ± 1.85	−78.03 ± 2.20	65.26 ± 2.43	−3.44 ± 0.03	−16.21 ± 1.85
3UG	0	−24.81 ± 0.56	−58.7 ± 0.61	−83.5 ± 0.55	72.45 ± 0.95	−2.93 ± 0.02	−13.99 ± 0.40
Bhft	0	−33.44 ± 0.70	−68.73 ± 1.72	−102.16 ± 0.92	96.57 ± 1.57	−3.73 ± 0.05	−9.33 ± 2.04
Ketoprofen glucuronide	−1	−35.30 ± 0.29	−183.62 ± 2.00	−218.92 ± 0.78	213.98 ± 0.88	−4.17 ± 0.01	−9.12 ± 0.55
Polmacoxib	0	−26.56 ± 0.45	−27.71 ± 2.57	−54.28 ± 1.15	54.70 ± 1.67	−3.40 ± 0.02	−2.98 ± 0.46
Bemetizide	0	−32.23 ± 1.69	−31.56 ± 3.37	−63.78 ± 2.21	68.27 ± 7.94	−3.61 ± 0.12	0.87 ± 2.80

Standard errors were computed with block averaging by dividing the trajectories into five blocks for improved statistics.

**Table 6:** Binding affinities (kcal/mol) between hCA family members with three selected inhibitors of 84Z, cyclothiazide, and 3UG from 50 replicates of docking predications with random seeds.

Name	Identifier	84Z	Cyclothiazide	3UG
hCA I	7Q0D	−8.0 ± 0.1	−9.1 ± 0.1	−7.9 ± 0.1
hCA II	1BCD	−8.0 ± 0.1	−8.3 ± 0.1	−7.4 ± 0.1
hCA III	3UYN	−6.8 ± 0.1	−7.5 ± 0.1	−6.8 ± 0.1
hCA IV	5IPZ	−6.9 ± 0.1	−6.5 ± 0.2	−6.2 ± 0.1
hCA VA	AF-P35218-F1	−6.2 ± 0.1	−6.9 ± 0.1	−5.8 ± 0.2
hCA VB	AF-Q9Y2D0-F1	−7.0 ± 0.1	−7.6 ± 0.2	−6.7 ± 0.1
hCA VI	3FE4	−6.0 ± 0.1	−7.1 ± 0.1	−6.1 ± 0.1
hCA VII	6H37	−7.2 ± 0.1	−8.1 ± 0.1	−7.4 ± 0.1
hCA IX	6FE1	−7.5 ± 0.1	−8.2 ± 0.1	−7.3 ± 0.1
hCA XII	1JD0	−7.1 ± 0.2	−7.6 ± 0.3	−6.9 ± 0.1
hCA XIII	4KNM	−7.8 ± 0.1	−8.9 ± 0.1	−7.9 ± 0.1
hCA XIV	4LU3	−7.8 ± 0.1	−8.3 ± 0.1	−7.3 ± 0.1

## Data Availability

Data are contained within the article.

## Supplementary Materials

The following supporting information can be downloaded at: https://www.mdpi.com/article/10.3390/ijms241612619/s1.
