# Supplementary material for: Repurposing of World-Approved Drugs for Potential Inhibition against Human Carbonic Anhydrase I: A Computational Study
Source: Int J Mol Sci. 2023 Aug 9;24(16):12619. doi: 10.3390/ijms241612619 (PMC10454238; doi:10.3390/ijms241612619)
Supplement: Supplementary file 1 [file ijms-24-12619-s001.zip › ijms-2513109-supplementary.pdf]

Supplementary Materials for

Repurposing of World-Approved Drugs for Potential Inhibition against Human Carbonic Anhydrase I: A Computational Study

Nannan Zheng [1], Wanyun Jiang [1], Puyu Zhang [1], Le Ma[1], Junzhao Chen[1] and Haiyang Zhang [1,*]

[1] Department of Biological Science and Engineering, School of Chemistry and Biological Engineering, University of Science and Technology Beijing, 100083 Beijing, China
* Correspondence: zhanghy@ustb.edu.cn

Table S1. Toxicity prediction of the existing ligand in complex with hCA I in the PDB database

Residue name	PDB ID	*q*	ΔE_{dock}	Toxicity				
				dili	carcino	immuno	mutagen	cyto
949	5GMM	0	-9.0	N(53)	N(62)	N(89)	N(70)	N(67)
TOR	3LXE	0	-8.5	N(66)	N(52)	N(84)	Y(52)	N(72)
GZE	6I0J	0	-8.5	Y(57)	N(61)	N(55)	N(62)	N(70)
V14	5E2M	0	-8.2	N(72)	N(72)	N(98)	N(72)	N(64)
BZE	6EVR	0	-8.1	N(78)	N(65)	N(99)	N(76)	N(63)
IWE	7ZL5	0	-8.0	N(59)	N(53)	N(95)	N(69)	N(62)
N19	6EX1	0	-8.0	N(80)	N(64)	N(99)	N(80)	N(61)
D3B	6FAF	0	-8.0	Y(54)	N(58)	N(99)	N(78)	N(78)
3UG	4WUQ	0	-8.0	N(66)	N(58)	N(99)	N(81)	N(69)
CJK	6F3B	0	-7.7	N(59)	N(64)	N(99)	N(79)	N(72)
O5N	6XZY	0	-7.7	N(65)	N(73)	N(90)	N(78)	N(68)
EON	6FAG	0	-7.6	Y(57)	N(57)	N(88)	N(73)	N(82)
O5H	6XZX	0	-7.5	N(70)	N(69)	N(99)	N(81)	N(64)
84Z	7Q0D	0	-7.4	N(65)	N(67)	N(99)	N(75)	N(70)
O4Z	6XZE	0	-7.3	N(65)	N(73)	N(93)	N(78)	N(68)
O5K	6XZS	0	-7.2	N(64)	N(72)	N(97)	N(82)	N(68)
3TV	4WR7	0	-7.1	N(75)	N(66)	N(98)	N(69)	N(78)
O55	6XZO	0	-7.0	N(65)	N(66)	Y(62)	N(74)	N(65)
7TI	7PLF	0	-6.9	N(74)	N(65)	N(95)	N(75)	N(76)
FLB	3W6I	0	-6.8	N(84)	N(53)	N(86)	N(76)	N(78)
O5T	6Y00	0	-6.7	N(65)	N(66)	Y(63)	N(74)	N(65)
M25	2NMX	0	-6.7	N(82)	N(73)	N(99)	N(79)	N(75)
M29	2NN7	0	-6.6	N(72)	N(64)	N(99)	N(69)	N(74)
M28	2NN1	0	-6.4	N(71)	N(67)	N(99)	N(73)	N(67)
AZM	3W6H	0	-6.3	N(56)	Y(51)	N(99)	N(85)	N(54)
MZM	1BZM	0	-6.3	N(81)	Y(50)	N(99)	N(70)	N(64)
AZM	1AZM	0	-5.9	N(56)	Y(51)	N(99)	N(85)	N(54)

3UF	4WUP	0	-5.7	N(82)	N(65)	N(99)	N(72)	N(75)
FO9	6G3V	0	-5.7	N(69)	Y(58)	N(99)	N(64)	N(54)
AAS	1CZM	0	-5.6	N(61)	N(62)	N(91)	N(80)	N(72)
GZH	6I0L	0	-5.5	N(64)	N(77)	N(91)	N(82)	N(77)
HIS	2FW4	1	-5.2	N(67)	N(80)	N(99)	N(66)	N(71)
PPF	2IT4	0	-4.3	N(94)	N(59)	N(99)	N(84)	N(70)
EDO	1JV0	0	-3.2	N(98)	N(86)	N(99)	N(94)	N(82)
BCT	1HCB	-1	-3.1	N(91)	N(63)	N(99)	N(88)	N(79)
EDO	1J9W	0	-1.7	N(98)	N(86)	N(99)	N(94)	N(82)

Table S2. Toxicity prediction of 79 compounds with ΔE_{dock} <= -9 kcal/mol and Zn^{2+}–ligand binding distances <= 3. 5 Å from word-approved drugs

**ZINC ID**	**Molecular structure**	***q***	**ΔE_{dock}**	**Toxicity**				
				**dili**	**carcino**	**immuno**	**mutagen**	**cyto**
ZINC000164760756	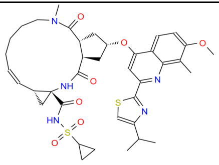	-1	-10.6	N(54)	N(59)	Y(92)	N(66)	N(52)
ZINC000003927200	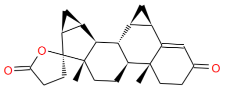	0	-10.4	N(79)	Y(66)	Y(89)	N(95)	N(83)
ZINC000060392779	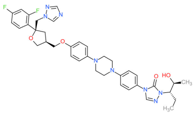	0	-10.2	Y(86)	N(62)	Y(99)	N(56)	N(75)
ZINC000012503149	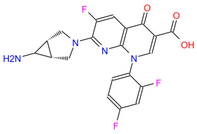	0	-10.2	Y(85)	Y(58)	Y(67)	Y(61)	N(89)
ZINC000100030989	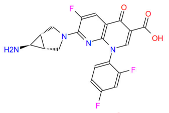	0	-10.2	Y(85)	Y(58)	Y(67)	Y(61)	N(89)
ZINC000147175374	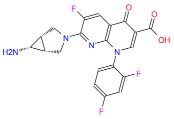	0	-10.2	Y(85)	Y(58)	Y(67)	Y(61)	N(89)
ZINC000095618351	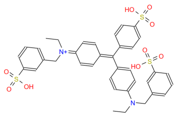	0	-10.1	N(70)	Y(53)	N(88)	Y(69)	N(58)
ZINC000003932831	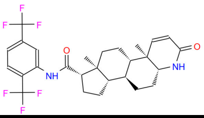	0	-10.1	N(92)	N(60)	Y(99)	N(64)	N(77)

ZINC0001 00067477	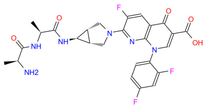	-1	-10	Y(85)	N(51)	N(57)	N(55)	N(70)
ZINC0001 00067477	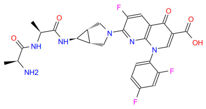	0	-10	Y(85)	N(51)	N(57)	N(55)	N(70)
ZINC0000 11679756	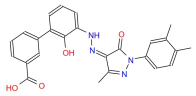	-3	-10	Y(67)	N(57)	N(72)	N(56)	N(84)
ZINC0000 11679756	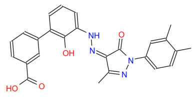	-2	-10	Y(67)	N(57)	N(72)	N(56)	N(84)
ZINC0000 14880001	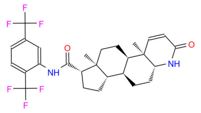	0	-10	N(92)	N(60)	Y(99)	N(64)	N(77)
ZINC0000 95618741	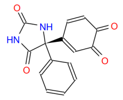	0	-9.9	Y(67)	Y(55)	N(98)	N(70)	N(66)
ZINC0000 11681563	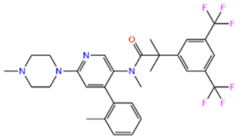	0	-9.9	N(66)	N(71)	N(78)	N(73)	N(64)
ZINC0000 11681563	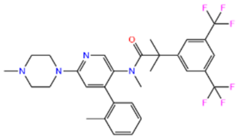	1	-9.9	N(66)	N(71)	N(78)	N(73)	N(64)
ZINC0009 36069565	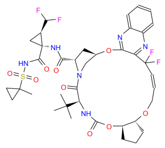	0	-9.9	Y(56)	N(58)	Y(96)	N(64)	N(50)
ZINC0000 04217252	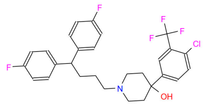	0	-9.9	N(94)	N(59)	Y(85)	N(83)	N(76)
ZINC0000 04217252	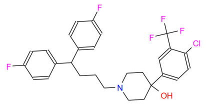	1	-9.9	N(94)	N(59)	Y(85)	N(83)	N(76)
ZINC0000 00601301	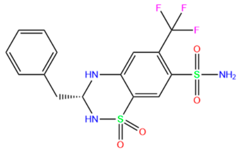	0	-9.8	N82)	N(71)	N(89)	N(84)	N73)
ZINC0002 05224698	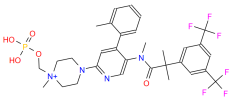	1	-9.8	N(66)	N(58)	N(53)	N(55)	N(61)
ZINC0000 04175630	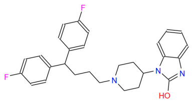	0	-9.8	N(78)	N(69)	Y(89)	N(86)	N(65)
ZINC0000 00538337	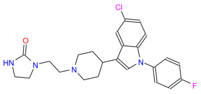	0	-9.8	N(75)	N(67)	Y(77)	N(56)	N(67)
ZINC0000 00538337	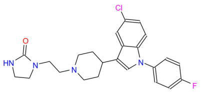	1	-9.8	N(75)	N(67)	Y(77)	N(56)	N(67)

ZINC000006716957	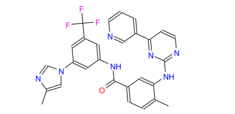	1	-9.7	Y(82)	Y(53)	Y(98)	N(59)	N(72)
ZINC000027990463	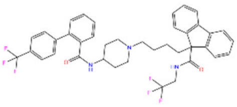	1	-9.7	N(78)	N(61)	N(70)	N(58)	N(77)
ZINC000000537755	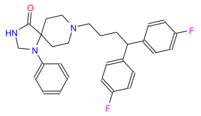	1	-9.7	N(74)	N(57)	Y(73)	N(69)	N(74)
ZINC000004175630	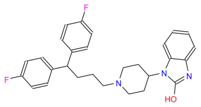	1	-9.7	N(78)	N(69)	Y(89)	N(86)	N(65)
ZINC000085599303	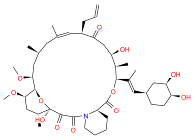	0	-9.7	N(83)	N(50)	Y(99)	N(73)	N(71)
ZINC000095618739	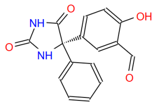	0	-9.6	Y(71)	Y(51)	N(96)	N(82)	N(74)
ZINC000685933136	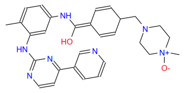	1	-9.6	N(55)	Y(52)	Y(80)	N(51)	N(63)
ZINC000021981256	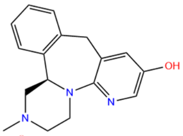	1	-9.6	N(83)	N(66)	N(96)	N(61)	N(59)
ZINC000003816514	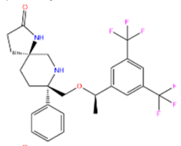	0	-9.6	N(83)	N(66)	N(93)	N(67)	N(75)
ZINC000003816514	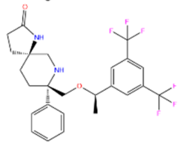	1	-9.6	N(83)	N(66)	N(93)	N(67)	N(75)
ZINC000261494552	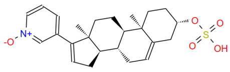	-1	-9.6	N(56)	N(68)	Y(82)	N(57)	N(65)
ZINC000006716957	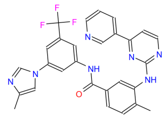	0	-9.5	Y(82)	Y(53)	Y(98)	N(59)	N(72)
ZINC000095618742	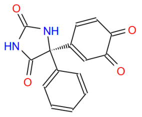	0	-9.5	Y(67)	Y(55)	N(98)	N(70)	N(66)
ZINC000011617039	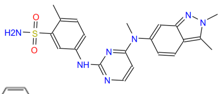	0	-9.5	N(65)	Y(66)	Y(85)	N(70)	Y(50)
ZINC000022034381	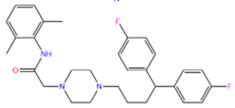	0	-9.5	N(79)	N(70)	N(97)	N(78)	N(67)
ZINC000095618852	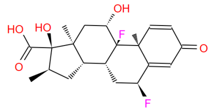	-1	-9.5	N(83)	Y(61)	Y(99)	N(76)	N(74)

ZINC0000 40165218	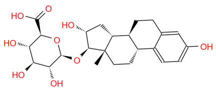	-1	-9.5	N(85)	N(66)	Y(99)	N(81)	N(64)
ZINC0002 61494710	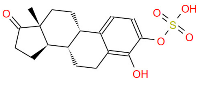	-1	-9.5	N(71)	N(77)	Y(82)	N(62)	N(78)
ZINC0002 57459143	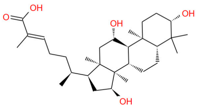	0	-9.5	N(58)	N(50)	Y(97)	N(71)	N(82)
ZINC0000 53073961	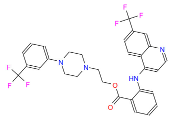	1	-9.4	N(78)	N(55)	Y(98)	N(65)	N(70)
ZINC0001 50338819	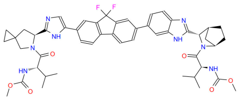	0	-9.4	N(63)	N(63)	Y(83)	N(67)	N(56)
ZINC0000 00020243	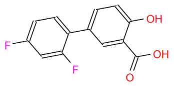	-1	-9.4	Y(75)	N(65)	N(97)	N(85)	N(60)
ZINC0001 00048501	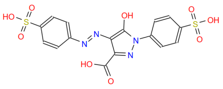	-4	-9.4	Y(51)	N(65)	N(99)	N(82)	N(66)
ZINC0000 03881958	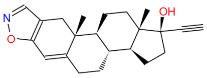	0	-9.4	Y(73)	N(60)	Y(78)	N(66)	N(76)
ZINC0000 31417974	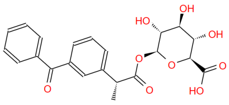	-1	-9.3	N(71)	N(68)	N(85)	N(85)	N(82)
ZINC0002 61494712	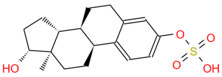	-1	-9.3	N(73)	N(78)	Y(99)	N(63)	N(75)
ZINC0000 31622970	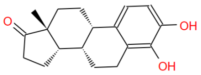	0	-9.3	N(70)	N(61)	Y(73)	N(89)	N(83)
ZINC0000 03873362	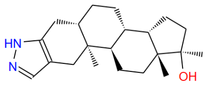	0	-9.3	N(53)	N(52)	Y(96)	N(67)	N(75)
ZINC0001 00051684	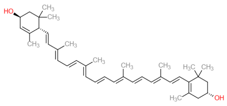	0	-9.3	N(79)	N(67)	Y(66)	N(81)	N(89)
ZINC0000 95862733	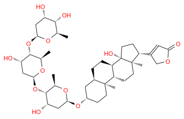	0	-9.3	N(96)	N(62)	Y(99)	N(93)	Y(59)
ZINC0001 00015190	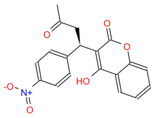	-1	-9.2	Y(51)	N(50)	N(95)	Y(85)	N(65)
ZINC0000 00601275	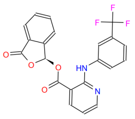	0	-9.2	Y(60)	Y(58)	N(52)	N(58)	N(70)
ZINC0000 02570882	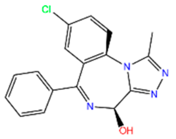	0	-9.2	N(52)	N(67)	N(97)	N(74)	N(70)

ZINC0000 04311748	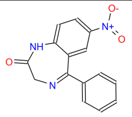	0	-9.2	N(50)	Y(61)	N(99)	N(65)	N(55)
ZINC0000 00607726	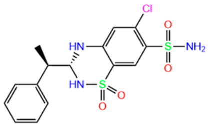	0	-9.2	N(89)	N(76)	N(88)	N(92)	N(81)
ZINC0000 22034381	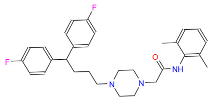	1	-9.2	N(79)	N(70)	N(97)	N(78)	N(67)
ZINC0000 01539579	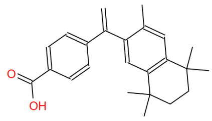	-1	-9.2	Y(80)	N(66)	N(99)	N(69)	N(79)
ZINC0000 95618879	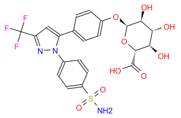	0	-9.2	Y(60)	Y(53)	N(70)	N(67)	N(79)
ZINC0001 18915338	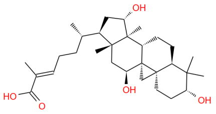	0	-9.2	N(58)	N(51)	Y(53)	N(60)	N(71)
ZINC0000 04026419	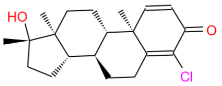	0	-9.2	Y(68)	Y(50)	Y(92)	N(92)	N(74)
ZINC0000 03873364	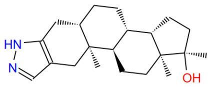	0	-9.2	N(53)	N(52)	Y(96)	N(67)	N(75)
ZINC0000 04215629	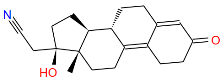	0	-9.2	Y(52)	N(72)	Y(61)	N(89)	N(81)
ZINC0000 00538152	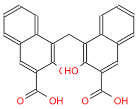	-2	-9.1	N(75)	N(71)	N(99)	N(62)	N(90)
ZINC0002 04073689	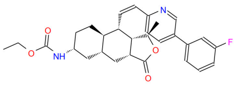	0	-9.1	N(62)	N(55)	Y(99)	N(58)	N(60)
ZINC0000 02017901	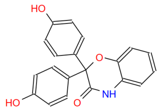	0	-9.1	Y(57)	Y(52)	N(99)	N(59)	N(69)
ZINC0000 00601254	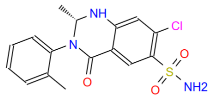	0	-9.1	N(65)	N(64)	N(73)	N(85)	Y(52)
ZINC0001 00053593	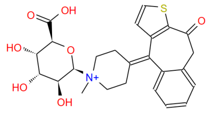	0	-9.1	N(71)	N(55)	N(82)	N(64)	N(51)
ZINC0000 43131420	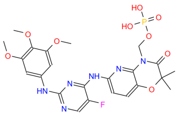	-2	-9.1	N(55)	N(65)	Y(99)	N(65)	N(61)
ZINC0000 85537142	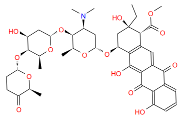	0	-9.1	N(71)	N(71)	Y(99)	Y(89)	N(57)

ZINC0000 85537142	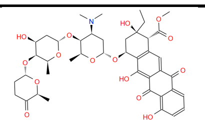	0	-9.1	N(71)	N(71)	Y(99)	Y(89)	N(57)
ZINC0000 33963989	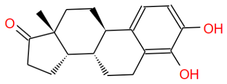	0	-9.1	N(70)	N(61)	Y(73)	N(89)	N(83)
ZINC0000 03875469	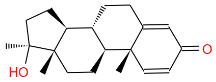	0	-9.1	Y(67)	N(56)	Y(97)	N(98)	N(89)
ZINC0002 61494708	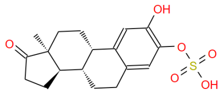	-1	-9.1	N(70)	N(76)	Y(98)	N(62)	N(78)
ZINC0001 00036907	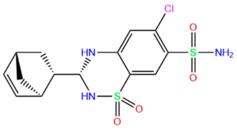	0	-9.1	N(91)	N(74)	N(91)	N(88)	N(73)
ZINC0002 42548690	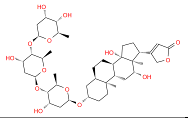	0	-9.1	N(98)	N(67)	Y(99)	N(59)	Y(59)

Table S3. Energy decomposition (kcal/mol) of identified key residues for binding with 84Z

Residue	ΔE_{vdW}	ΔE_{elec}	ΔE_{MM}	ΔG_{sol}	ΔE_{bind}
HIS-64	-0.1 ± -0.0	0.3 ± -0.1	0.3 ± -0.1	-0.4 ± -0.1	-0.1 ± -0.1
HIS-67	-0.1 ± -0.1	-0.1 ± -0.1	-0.2 ± -0.1	0.1 ± -0.1	-0.1 ± -0.1
GLN-92	-1.2 ± -0.3	0.6 ± -0.8	-0.6 ± -0.9	-1.1 ± -0.8	-1.7 ± -0.8
HIS-94	-1.3 ± -0.3	1.6 ± -0.8	0.3 ± -0.8	-2.1 ± -0.9	-1.8 ± -0.9
HIS-96	-0.2 ± -0.0	1.5 ± -0.2	1.3 ± -0.2	-2.5 ± -0.4	-1.3 ± -0.5
GLU-106	-0.3 ± -0.1	1.7 ± -1.1	1.3 ± -1.1	0.8 ± -1.9	2.2 ± -1.9
HIS-107	-0.1 ± -0.0	-1.9 ± -0.4	-2.0 ± -0.4	1.3 ± -0.5	-0.7 ± -0.5
GLU-117	-0.0 ± -0.0	1.9 ± -0.3	1.9 ± -0.3	-1.9 ± -0.4	0.0 ± -0.4
HIS-119	-0.9 ± -0.3	3.1 ± -0.6	2.1 ± -0.8	-3.6 ± -1.1	-1.5 ± -1.3
ALA-121	-0.9 ± -0.2	-0.6 ± -0.2	-1.5 ± -0.3	0.9 ± -0.4	-0.5 ± -0.4
HIS-122	-0.2 ± -0.1	-1.9 ± -0.3	-2.1 ± -0.3	3.1 ± -0.8	1.0 ± -0.7
LEU-131	-0.3 ± -0.2	-0.1 ± -0.1	-0.4 ± -0.3	0.1 ± -0.1	-0.3 ± -0.2
ALA-132	-0.0 ± -0.0	-0.1 ± -0.0	-0.1 ± -0.0	0.1 ± -0.0	-0.0 ± -0.0
ALA-135	-0.3 ± -0.2	-0.2 ± -0.2	-0.6 ± -0.3	0.4 ± -0.4	-0.1 ± -0.2
LEU-141	-1.3 ± -0.4	-0.5 ± -0.3	-1.8 ± -0.6	0.8 ± -0.4	-1.0 ± -0.4
VAL-143	-1.3 ± -0.5	-0.1 ± -0.1	-1.4 ± -0.4	0.5 ± -0.1	-0.9 ± -0.5
LEU-198	-2.2 ± -0.4	-0.0 ± -0.2	-2.3 ± -0.5	0.5 ± -0.2	-1.8 ± -0.5
THR-199	-0.5 ± -0.5	-3.5 ± -0.9	-4.0 ± -0.7	1.3 ± -0.4	-2.7 ± -0.6
HIS-200	-1.3 ± -0.7	-3.5 ± -1.5	-4.8 ± -1.2	2.4 ± -0.6	-2.3 ± -1.3
PRO-201	-0.1 ± -0.0	-0.4 ± -0.1	-0.5 ± -0.1	0.3 ± -0.1	-0.1 ± -0.1
PRO-202	-0.2 ± -0.1	0.2 ± -0.1	0.0 ± -0.1	-0.1 ± -0.0	-0.1 ± -0.1
TRP-209	-0.8 ± -0.3	-1.8 ± -1.1	-2.7 ± -1.1	1.1 ± -0.5	-1.5 ± -0.8
ZN-261	0.9 ± -0.6	-20.3 ± -2.8	-19.4 ± -2.4	38.7 ± -5.2	19.3 ± -5.7

Table S4. Energy decomposition (kcal/mol) of identified key residues for binding with cyclothiazide

Residue	ΔE_{vdW}	ΔE_{elec}	ΔE_{MM}	ΔG_{sol}	ΔE_{bind}
HIS-64	-0.8 ± -0.4	-0.6 ± -1.6	-1.4 ± -1.6	0.9 ± -1.0	-0.5 ± -0.8
HIS-67	-1.5 ± -0.5	-0.6 ± -0.9	-2.2 ± -1.2	1.0 ± -0.7	-1.2 ± -1.1
GLN-92	-1.5 ± -0.5	-0.3 ± -1.0	-1.8 ± -1.0	0.0 ± -0.7	-1.8 ± -0.8
HIS-94	-2.0 ± -0.4	1.5 ± -0.8	-0.5 ± -1.0	-2.5 ± -1.4	-3.0 ± -1.7
HIS-96	-0.3 ± -0.0	1.3 ± -0.3	1.1 ± -0.3	-3.4 ± -1.3	-2.3 ± -1.3
GLU-106	-0.1 ± -0.0	3.0 ± -0.5	2.9 ± -0.5	-4.2 ± -1.1	-1.3 ± -1.1
HIS-107	-0.0 ± -0.0	-2.2 ± -0.2	-2.2 ± -0.2	2.5 ± -0.5	0.3 ± -0.5
GLU-117	-0.0 ± -0.0	2.2 ± -0.2	2.2 ± -0.2	-2.9 ± -0.5	-0.7 ± -0.5
HIS-119	-0.7 ± -0.1	3.2 ± -0.6	2.5 ± -0.6	-5.5 ± -1.1	-3.0 ± -1.1
ALA-121	-0.4 ± -0.1	-0.1 ± -0.1	-0.5 ± -0.1	0.4 ± -0.1	-0.1 ± -0.1
HIS-122	-0.1 ± -0.0	-0.1 ± -0.3	-0.2 ± -0.3	0.6 ± -0.3	0.4 ± -0.2
LEU-131	-0.1 ± -0.0	0.1 ± -0.1	-0.0 ± -0.1	-0.1 ± -0.1	-0.1 ± -0.0
ALA-132	-0.0 ± -0.0	-0.0 ± -0.0	-0.0 ± -0.0	0.0 ± -0.0	-0.0 ± -0.0
ALA-135	-0.1 ± -0.0	0.2 ± -0.1	0.1 ± -0.1	-0.2 ± -0.1	-0.1 ± -0.1
LEU-141	-0.4 ± -0.1	0.1 ± -0.1	-0.4 ± -0.1	0.2 ± -0.1	-0.2 ± -0.1
VAL-143	-0.7 ± -0.2	-0.2 ± -0.1	-0.9 ± -0.2	0.3 ± -0.1	-0.6 ± -0.2
LEU-198	-1.8 ± -0.3	-0.4 ± -0.3	-2.2 ± -0.5	0.3 ± -0.2	-1.9 ± -0.5
THR-199	-0.7 ± -0.3	-2.0 ± -0.9	-2.7 ± -0.7	0.7 ± -0.5	-2.0 ± -0.7
HIS-200	-2.1 ± -0.6	-4.2 ± -0.7	-6.4 ± -0.7	4.2 ± -0.5	-2.2 ± -0.8
PRO-201	-0.4 ± -0.1	-0.5 ± -0.2	-0.9 ± -0.2	0.9 ± -0.3	0.0 ± -0.2
PRO-202	-0.4 ± -0.1	0.2 ± -0.1	-0.2 ± -0.1	-0.1 ± -0.1	-0.3 ± -0.1
TRP-209	-0.8 ± -0.2	0.1 ± -0.3	-0.8 ± -0.3	0.5 ± -0.1	-0.3 ± -0.3
ZN-261	0.4 ± -0.5	-20.4 ± -3.6	-19.9 ± -3.2	46.9 ± -10.7	26.9 ± -10.1

Table S5. Energy decomposition (kcal/mol) of identified key residues for binding with 3UG

**Residue**	**ΔE_{vdW}**	**ΔE_{elec}**	**ΔE_{MM}**	**ΔG_{sol}**	**ΔE_{bind}**
HIS-64	-0.1 ± -0.0	0.6 ± -0.1	0.5 ± -0.1	-0.7 ± -0.1	-0.2 ± -0.1
HIS-67	-0.1 ± -0.0	-0.1 ± -0.1	-0.2 ± -0.1	0.2 ± -0.1	-0.0 ± -0.1
GLN-92	-0.9 ± -0.3	1.5 ± -0.5	0.5 ± -0.5	-1.5 ± -0.6	-1.0 ± -0.4
HIS-94	-0.2 ± -0.5	5.4 ± -1.2	5.2 ± -1.3	-7.3 ± -1.1	-2.1 ± -1.3
HIS-96	-0.5 ± -0.1	4.5 ± -0.6	3.9 ± -0.6	-7.1 ± -0.7	-3.1 ± -0.6
GLU-106	-0.1 ± -0.0	5.0 ± -0.6	4.9 ± -0.5	-6.4 ± -0.8	-1.6 ± -0.8
HIS-107	-0.0 ± -0.0	-2.9 ± -0.2	-2.9 ± -0.2	4.3 ± -0.4	1.3 ± -0.4
GLU-117	-0.0 ± -0.0	3.1 ± -0.2	3.0 ± -0.2	-4.7 ± -0.4	-1.6 ± -0.4
HIS-119	-0.8 ± -0.1	5.3 ± -0.7	4.5 ± -0.8	-8.4 ± -0.7	-3.9 ± -0.7
ALA-121	-0.4 ± -0.1	-0.1 ± -0.0	-0.5 ± -0.1	0.7 ± -0.3	0.2 ± -0.2
HIS-122	-0.1 ± -0.0	-0.4 ± -0.2	-0.4 ± -0.2	1.5 ± -0.6	1.1 ± -0.5
LEU-131	-0.6 ± -0.3	-0.0 ± -0.1	-0.7 ± -0.2	-0.0 ± -0.2	-0.7 ± -0.3
ALA-132	-0.1 ± -0.0	-0.1 ± -0.0	-0.1 ± -0.0	0.0 ± -0.0	-0.1 ± -0.0
ALA-135	-0.5 ± -0.2	0.1 ± -0.1	-0.4 ± -0.2	-0.3 ± -0.2	-0.7 ± -0.3
LEU-141	-0.8 ± -0.2	0.1 ± -0.1	-0.7 ± -0.2	0.6 ± -0.2	-0.2 ± -0.3
VAL-143	-0.6 ± -0.1	-0.3 ± -0.1	-0.8 ± -0.2	0.5 ± -0.1	-0.4 ± -0.2
LEU-198	-2.0 ± -0.5	-0.8 ± -0.2	-2.9 ± -0.5	1.4 ± -0.2	-1.5 ± -0.5
THR-199	-0.8 ± -0.1	-0.6 ± -0.4	-1.4 ± -0.4	1.5 ± -0.3	0.1 ± -0.4
HIS-200	-0.8 ± -0.7	-3.4 ± -0.9	-4.2 ± -0.7	2.2 ± -0.6	-2.1 ± -0.6
PRO-201	-0.3 ± -0.1	-0.1 ± -0.1	-0.4 ± -0.1	0.8 ± -0.3	0.3 ± -0.3
PRO-202	-0.7 ± -0.3	0.1 ± -0.1	-0.6 ± -0.3	0.2 ± -0.1	-0.4 ± -0.3
TRP-209	-0.6 ± -0.2	-0.5 ± -0.1	-1.1 ± -0.2	0.6 ± -0.1	-0.4 ± -0.2
ZN-261	1.8 ± -0.8	-45.8 ± -4.3	-44.0 ± -3.8	77.0 ± -4.7	33.0 ± -5.0

Table S6. Energy decomposition (kcal/mol) of identified key residues for binding with ketoprofen glucuronide

Residue	ΔE_{vdW}	ΔE_{elec}	ΔE_{MM}	ΔG_{sol}	ΔE_{bind}
HIS-64	-0.2 ± -0.1	-1.6 ± -0.3	-1.8 ± -0.3	1.4 ± -0.3	-0.4 ± -0.1
HIS-67	-0.2 ± -0.0	0.1 ± -0.5	-0.1 ± -0.5	-0.0 ± -0.4	-0.1 ± -0.2
GLN-92	-1.4 ± -0.2	1.0 ± -0.7	-0.4 ± -0.6	-0.3 ± -0.7	-0.8 ± -0.4
HIS-94	-0.5 ± -0.4	11.0 ± -1.0	10.5 ± -1.2	-9.5 ± -1.0	1.0 ± -1.1
HIS-96	-0.3 ± -0.0	8.4 ± -0.4	8.2 ± -0.4	-9.6 ± -0.9	-1.4 ± -0.8
GLU-106	-0.1 ± -0.0	24.6 ± -0.9	24.6 ± -0.9	-21.9 ± -1.0	2.6 ± -1.0
HIS-107	-0.0 ± -0.0	-18.9 ± -0.6	-18.9 ± -0.6	17.1 ± -0.7	-1.7 ± -0.5
GLU-117	-0.0 ± -0.0	19.5 ± -0.5	19.5 ± -0.5	-18.0 ± -0.7	1.5 ± -0.5
HIS-119	-0.7 ± -0.1	11.9 ± -1.2	11.2 ± -1.3	-11.2 ± -1.0	-0.0 ± -1.1
ALA-121	-1.2 ± -0.2	-1.6 ± -0.2	-2.8 ± -0.4	1.6 ± -0.4	-1.2 ± -0.4
HIS-122	-0.5 ± -0.1	-14.7 ± -0.5	-15.2 ± -0.5	16.3 ± -0.7	1.1 ± -0.6
LEU-131	-2.0 ± -0.3	0.5 ± -0.1	-1.4 ± -0.3	-0.2 ± -0.3	-1.6 ± -0.4
ALA-132	-0.7 ± -0.2	0.4 ± -0.1	-0.3 ± -0.3	-0.2 ± -0.1	-0.6 ± -0.3
ALA-135	-1.1 ± -0.2	0.8 ± -0.2	-0.3 ± -0.2	-0.5 ± -0.1	-0.8 ± -0.2
LEU-141	-1.1 ± -0.2	-1.2 ± -0.2	-2.4 ± -0.3	0.9 ± -0.2	-1.4 ± -0.3
VAL-143	-0.6 ± -0.1	-1.5 ± -0.3	-2.1 ± -0.3	1.3 ± -0.2	-0.8 ± -0.2
LEU-198	-1.8 ± -0.3	-3.2 ± -0.3	-5.0 ± -0.5	2.8 ± -0.2	-2.2 ± -0.5
THR-199	-0.3 ± -0.1	-1.0 ± -0.5	-1.4 ± -0.5	2.1 ± -0.4	0.7 ± -0.4
HIS-200	-1.2 ± -0.5	2.4 ± -1.1	1.1 ± -1.0	-0.3 ± -0.5	0.8 ± -0.8
PRO-201	-0.3 ± -0.1	1.1 ± -0.4	0.8 ± -0.4	-0.4 ± -0.4	0.4 ± -0.3
PRO-202	-0.8 ± -0.2	-0.9 ± -0.2	-1.7 ± -0.4	0.7 ± -0.2	-1.0 ± -0.3
TRP-209	-0.6 ± -0.1	-0.9 ± -0.5	-1.4 ± -0.5	1.1 ± -0.3	-0.3 ± -0.3
ZN-261	2.8 ± -1.0	-124.1 ± -3.8	-121.3 ± -3.0	131.0 ± -8.0	9.8 ± -7.8

Table S7. Energy decomposition (kcal/mol) of identified key residues for binding with bhft

**Residue**	**ΔE_{vdW}**	**ΔE_{elec}**	**ΔE_{MM}**	**ΔG_{sol}**	**ΔE_{bind}**
HIS-64	-0.1 ± -0.1	0.3 ± -0.2	0.1 ± -0.2	-0.3 ± -0.2	-0.2 ± -0.2
HIS-67	-0.3 ± -0.1	0.3 ± -0.2	-0.0 ± -0.3	-0.2 ± -0.5	-0.3 ± -0.3
GLN-92	-1.2 ± -0.4	-0.2 ± -0.7	-1.4 ± -0.9	1.2 ± -0.8	-0.2 ± -0.7
HIS-94	-0.6 ± -0.5	8.0 ± -1.2	7.4 ± -1.3	-9.7 ± -1.0	-2.3 ± -1.1
HIS-96	-0.5 ± -0.1	4.4 ± -0.5	3.9 ± -0.5	-7.1 ± -0.7	-3.2 ± -0.6
GLU-106	-0.1 ± -0.0	5.4 ± -0.6	5.3 ± -0.6	-6.1 ± -1.6	-0.8 ± -1.7
HIS-107	-0.0 ± -0.0	-3.2 ± -0.2	-3.2 ± -0.2	4.1 ± -0.6	0.9 ± -0.6
GLU-117	-0.0 ± -0.0	3.5 ± -0.2	3.5 ± -0.2	-4.8 ± -0.6	-1.4 ± -0.6
HIS-119	-1.0 ± -0.1	5.9 ± -0.7	4.9 ± -0.8	-8.3 ± -0.8	-3.3 ± -1.0
ALA-121	-0.3 ± -0.2	-0.0 ± -0.1	-0.3 ± -0.2	0.3 ± -0.1	-0.0 ± -0.2
HIS-122	-0.1 ± -0.0	0.1 ± -0.2	0.1 ± -0.2	0.6 ± -0.3	0.7 ± -0.3
LEU-131	-1.3 ± -0.5	-0.2 ± -0.4	-1.5 ± -0.7	0.8 ± -0.7	-0.7 ± -0.5
ALA-132	-0.8 ± -0.4	-0.0 ± -0.2	-0.8 ± -0.4	0.1 ± -0.2	-0.7 ± -0.4
ALA-135	-0.6 ± -0.2	0.5 ± -0.1	-0.1 ± -0.2	-0.4 ± -0.2	-0.6 ± -0.2
LEU-141	-0.7 ± -0.2	0.1 ± -0.1	-0.6 ± -0.2	0.3 ± -0.1	-0.2 ± -0.2
VAL-143	-0.6 ± -0.2	-0.3 ± -0.1	-0.9 ± -0.2	0.6 ± -0.1	-0.3 ± -0.3
LEU-198	-2.5 ± -0.3	-0.8 ± -0.2	-3.2 ± -0.4	1.4 ± -0.3	-1.8 ± -0.5
THR-199	-0.8 ± -0.1	-0.0 ± -0.4	-0.8 ± -0.4	1.1 ± -0.3	0.3 ± -0.5
HIS-200	-1.2 ± -0.7	-5.2 ± -1.0	-6.5 ± -0.8	4.2 ± -0.7	-2.2 ± -0.8
PRO-201	-0.4 ± -0.1	0.5 ± -0.3	0.1 ± -0.3	0.6 ± -0.4	0.7 ± -0.5
PRO-202	-0.7 ± -0.2	0.0 ± -0.2	-0.6 ± -0.2	0.1 ± -0.1	-0.5 ± -0.2
TRP-209	-0.7 ± -0.2	-0.4 ± -0.1	-1.1 ± -0.2	0.7 ± -0.2	-0.4 ± -0.2
ZN-261	2.2 ± -1.1	-51.3 ± -4.0	-49.1 ± -3.3	83.9 ± -5.0	34.8 ± -5.2

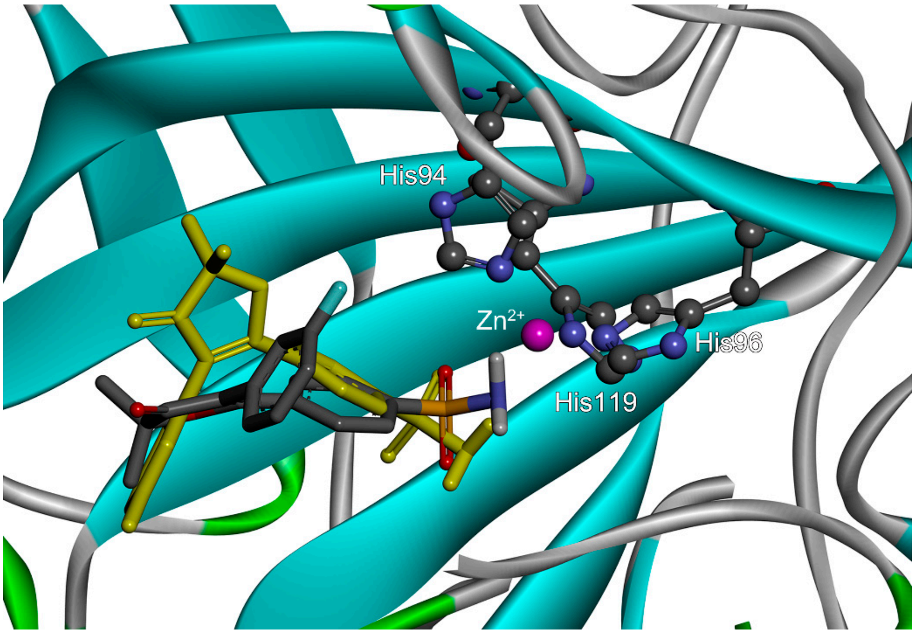

Figure S1. Comparison of crystal structure with docking pose for the binding of polmacoxib to hCA I. Crystal structure was taken from PDB database (PDB code: 5GMM). The crystal ligand was colored by element, and the docking pose was in yellow.

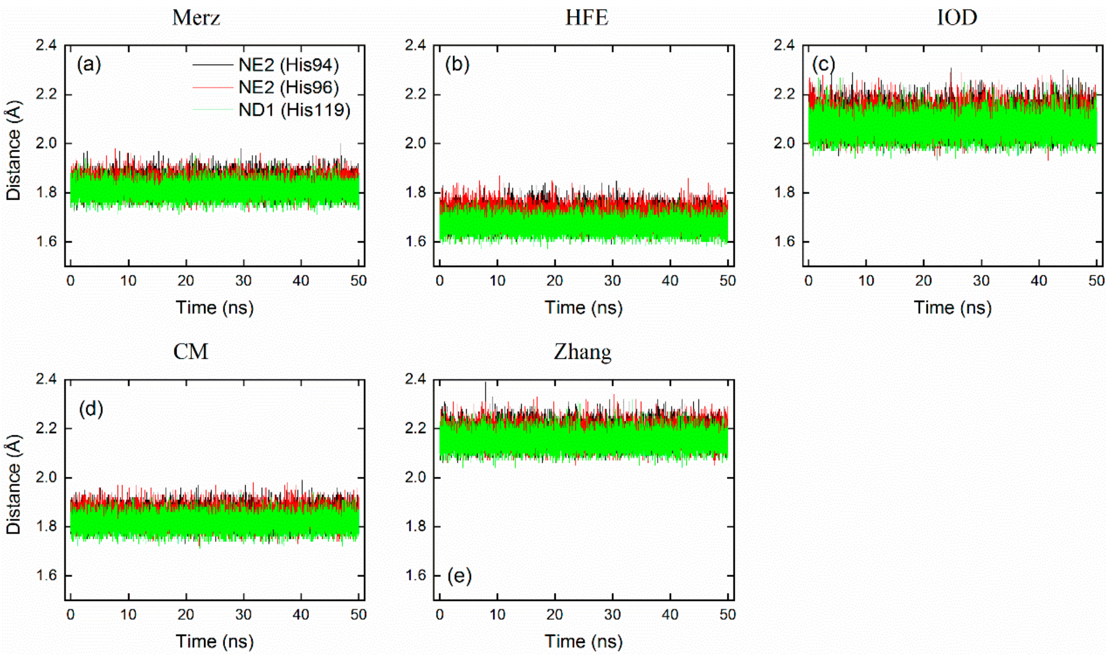

Figure S2. Zn^{2+}–ligand binding distance as a function of simulation time for ligand-free hCA I using modified Amber ff14SB protein force fields with different ion models (a-e). Refer to Table 3 in the main text for the details of force field parameters. Distances with the NE2 atom (black) of His94, the NE2 atom (red) of His96, and the ND1 atom (green) of His119 were monitored.

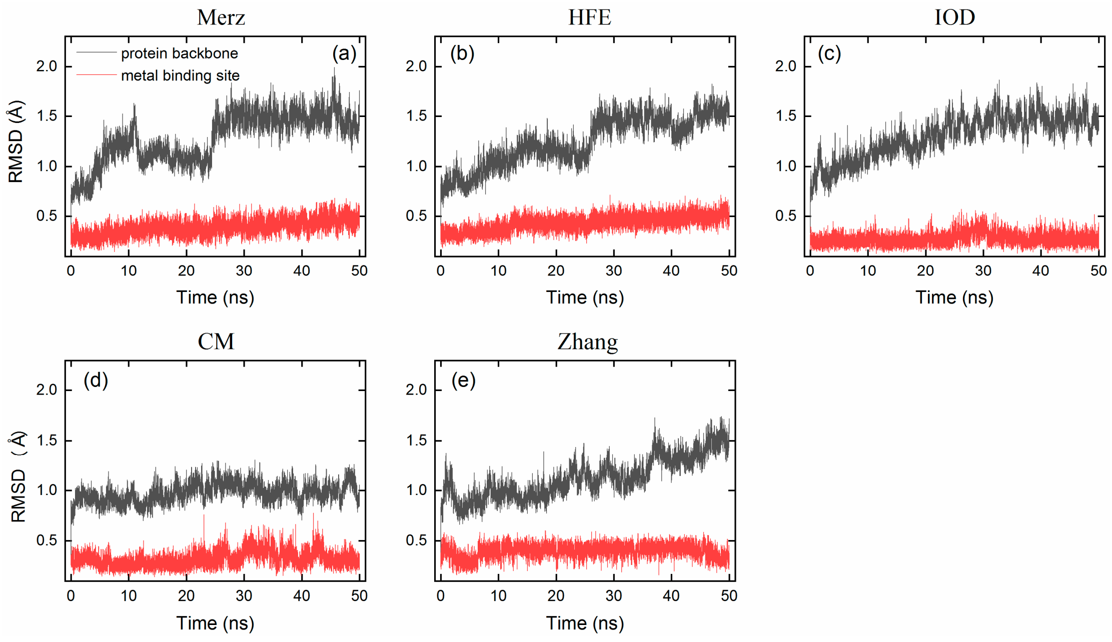

Figure S3. RMSD of hCA I protein backbone (black) and metal binding site (red) from crystal structure as a function of simulation time for ligand-free hCA I using modified Amber ff14SB protein force fields with different ion models (a-e). Refer to Table 3 for the details of force field parameters.
